# Arteriovenous Fistula after Mandibular Fracture: A Case Report

**DOI:** 10.29252/wjps.9.3.343

**Published:** 2020-09

**Authors:** Gholamreza Motazedian, Ali Khojasteh

**Affiliations:** Department of Plastic and Reconstructive Surgery, Shiraz University of Medical Sciences, Shiraz, Iran

**Keywords:** Arteriovenous fistula, Mandibular fracture

## Abstract

We described a rare case of arteriovenous (AV) fistula after mandibular fracture in a 64-year-old man with chronic schizophrenia. The diagnosis was made by CT angiography. The patient suffered two episodes of mandibular fracture 3 months and 12 months ago. He was found to have a large AV fistula in left side of his neck. So the patient was scheduled for operation to correct fistula.

## INTRODUCTION

Mandibular fractures are the most common fractures of the facial skeleton.^1^ The fractures result in severe loss of function and disfigurement.^2^ Mandible, being the only mobile bone of facial skeleton plays a major role in mastication, speech and deglutition.^3^ The most common location is the angle of mandible^4^ and the prominent causes of fracture mandible include road traffic accidents, falls, interpersonal violence and sports injuries.^5^ In mandibular fracture, AV fistula is a rare condition that could be fatal if left untreated as the result of massive blood loss after tooth extraction or attempts to remove or biopsy the lesion.^6^ Herein, we presented a patient with AV fistula after mandibular fracture.

## CASE REPORT

**Fig. 1:**
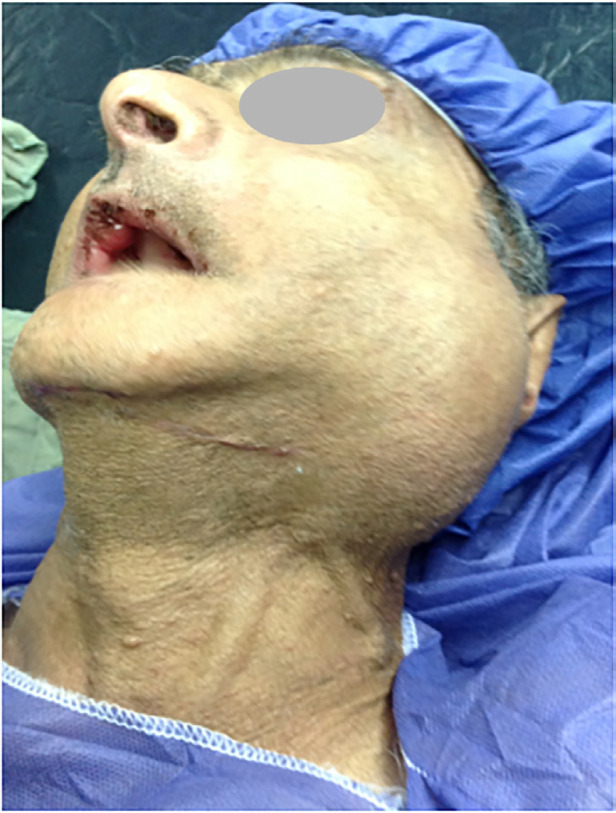
A 64-year-old man with a history of two episodes of mandibular fracture 3 months and 12 months ago was referred to the trauma center with chief complaint of left side neck bulging

**Fig. 2:**
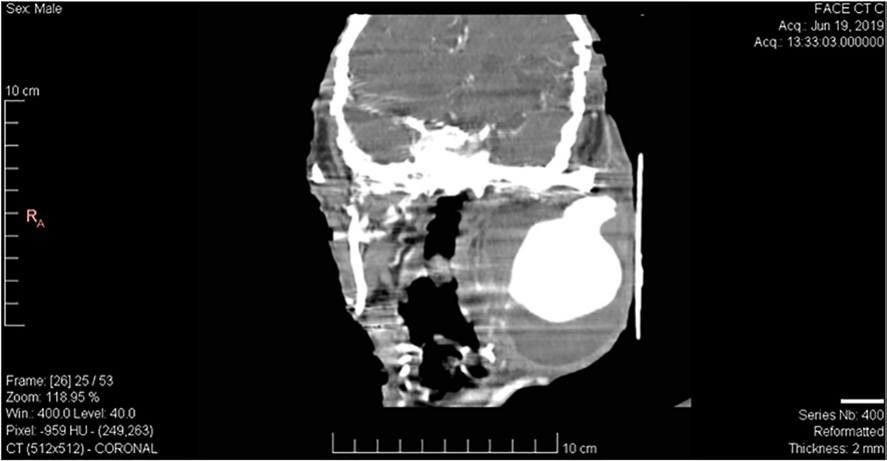
CT angiography shows a large lesion in left side of neck from left external carotid artery supplying the enhancing mandibular arteriovenous fistula

A 64-year-old man known as a case of chronic schizophrenia with a history of two episodes of mandibular fracture 3 months and 12 months ago was referred to our trauma center with chief complaint of left side neck bulging (Figure 1). A CT angiography was conducted which showed a large AV fistula in the left side of the neck (Figure 2). The patient was scheduled for operation to correct the fistula. In this patient with edentulous condition, chronic schizophrenia and severe disequilibrium were visible and he suffered from two episodes of mandibular fracture in the right and the left side of the mandibular body as a bone spur was the cause of AV fistula. His previous mandibular fractures were treated extra-orally and we were cautious about drilling direction to fix the fractures.

**Fig. 3:**
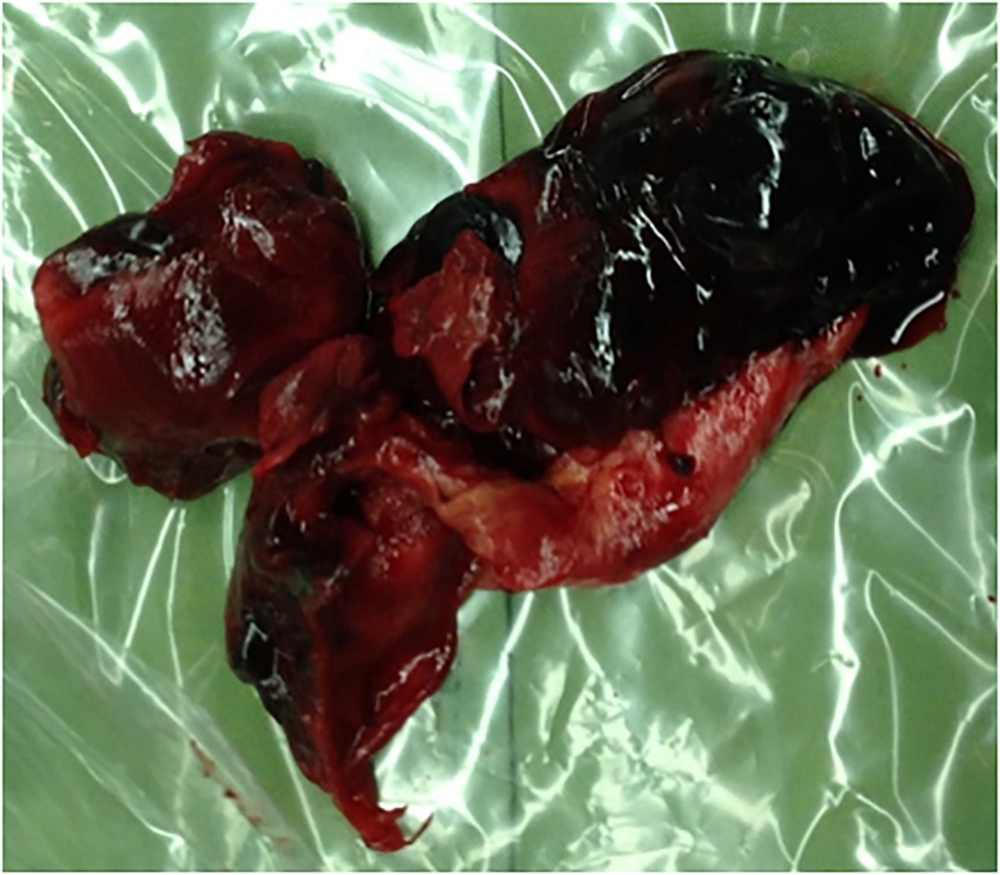
A pseudo-aneurysm was detected between left external carotid branch and left facial vein that was excised

**Fig. 4:**
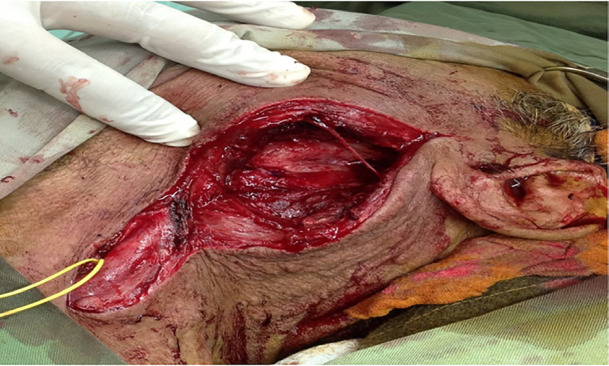
The left facial vein was ligated and perforation of the left external carotid branch was repaired with prolene 6-0

Under general anesthesia, in proper position, an incision was made on the left side of the neck. Proximal control was applied on the common carotid using an umbilical tape. Then aneurysmal mass was opened meticulously. Large clot was evacuated and a pseudo-aneurysm was detected between left external carotid branch and left facial vein that was excised (Figure 3). The left facial vein was ligated and any perforation of the left external carotid branch was repaired with prolene 6-0 (Figure 4). The wound was closed and the patient was discharged uneventfully. All procedures performed were in accordance with the ethical standards of the institutional and national research committee and with the 1964 Helsinki declaration and its later amendments or comparable ethical standards. Informed consent was obtained from the patient

## DISCUSSION

Trauma to the maxillofacial area produces a variety of injuries. These injuries may be simple and limited to the soft tissues or they may be complex and involve multiple facial bones.^7^

Mandible is the second most fractured bone in the whole body. It may fracture alone or in combination with other facial bones.^8^ Different complications have been reported after mandibular fractures. The most common ones are infection, malocclusion, malunion, nonunion, TMJ dysfunction and nerve damage. AV fistula on the other hand is a rare complication after mandibular fracture that could be fatal if left untreated as the result of massive blood loss after tooth extraction or attempts to remove or biopsy the lesion.^6^

AV fistula may be due to a sharp bone penetration to adjacent vessels. In some cases, the vein and artery are damaged and the healing process would result in AV fistula. The main symptom of AV fistula is a mass effect near the skin surface. The role of CT in the diagnosis of AV fistula has been greatly improved by the introduction of CT angiography. Different complications have been reported after mandibular fractures. The most common ones are infection, malocclusion, malunion, nonunion, TMJ dysfunction and nerve damage.

## CONCLUSION

AV fistula is a rare complication after mandibular fracture that should be diagnosed and treated properly.
